# New Synthesis Method for Sultone Derivatives: Synthesis, Crystal Structure and Biological Evaluation of S-CA

**DOI:** 10.3390/molecules20034307

**Published:** 2015-03-06

**Authors:** Bi Li, Wenqiang Yan, Chenze Zhang, Yuzhong Zhang, Miao Liang, Fuhao Chu, Yan Gong, Bing Xu, Penglong Wang, Haimin Lei

**Affiliations:** 1 School of Chinese Pharmacy, Beijing University of Chinese Medicine, Beijing 100102, China; E-Mails: libimegan@163.com (B.L.); ywq3226925@163.com (W.Y.); zcz920418@163.com (C.Z); mimi1111202@sina.com (M.L.); chufhao@163.com (F.C.); gongyan90@163.com (Y.G.); weichenxubing@126.com (B.X.); 2 School of Basic Medicine, Beijing University of Chinese Medicine, Beijing 100029, China; E-Mail: zyz100102@126.com

**Keywords:** sultone derivative, ring-closing reaction, CAM, acute toxicity, S180

## Abstract

There has been no remarkable progress in the synthesis of sultones in recent years. To facilitate more detailed studies of this functional group, we found a new method to synthesize the sulfonic acid lactone derivatives and finish its ring-closing reaction. A new sultone derivative, (*E*)-ethyl 4-oxo-6-styryl-3,4-dihydro-1,2-oxathiine-5-carboxylate 2,2-dioxide (**S-CA**), was synthesized and structurally identified by ^1^H-NMR, ^13^C-NMR, HMQC and X-ray single crystal diffraction analysis. The new rapid synthesis extended the method of ring-closing reaction of sulfonic acid lactone derivatives. The angiogenesis activities of **S-CA** were evaluated by the chick chorioallantoic membrane (CAM) model. It could selectively suppress small angiogenesis in CAM, without influencing either middle and large angiogenesis. In addition, anticancer efficacy of **S-CA** was evaluated *in vivo* using a murine sarcoma S180 model. Reduction of the tumor weight and tumor HE staining regions demonstrated that **S-CA** (10 mg/kg, intraperitoneal injection) had potent inhibition effects and a 44.71% inhibitory rate in S180 mice. Moreover, an acute toxicity test showed that the LD_50_ value of **S-CA** via intraperitoneal injection was 25.624 mg/kg.

## 1. Introduction

The importance of the sultone unit in organic chemistry cannot be overstated. Since the term “sultone” was first introduced into the literature by Erdmann in 1888, it has emerged as valuable heterocyclic intermediate [1,2,3,4]; sultones undergo cleavage of their carbon–oxygen bond in the presence of nucleophiles and can be used as sulfoalkylating agents in both general organic synthesis and natural product synthesis [5,6]. Methods used to synthesize sultones and include intramolecular Diels–Alder reactions, ring-closing metathesis, Pd-catalyzed intramolecular coupling reactions, Rh-catalyzed C–H insertion, Rh-catalyzed carbene cyclization cycloaddition cascade reactions, nucleophilic addition reactions *etc.* [7,8]. The major step in synthesis of sultone is ring-closing reaction which was often complicated by the need to use special and expensive catalysts such as Rh and Pd [8,9,10]. However, the search for a simple and short synthesis of sultones has never been suspended for a moment in recent years [11]. To facilitate the synthesis of this functional group, in present work, we found a new method to synthesize sulfonic acid lactone derivatives and perform their ring-closing reactions. The simple and rapid synthesis method we have found has never been reported previously. The newly synthesized compound **S-CA** was characterized by ^1^H-NMR, ^13^C-NMR, HMQC and X-ray single crystal diffraction analysis.

The biological activities of sultones previously included toxicological, skin sensitization, antiviral (anti-HIV and HCMV) and antitumor activities [12,13,14]. Some compounds prepared by total synthesis had antitumor activities due to their sulphonic acid ester structures [15,16]. Therefore, in this article an angiogenesis evaluation on CAM and antitumor test on murine sarcoma S180 of **S-CA** were investigated. Moreover, an acute toxicity test, as a part of safety evaluation of **S-CA**, was carried out via intraperitoneal injection in Kunming mice to investigate its potential toxicity.

## 2. Results and Discussion

### 2.1. Chemistry

#### 2.1.1. Synthesis of **S-CA**

**Scheme 1:**
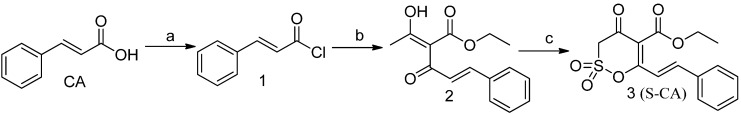
The synthetic route to **S-CA**.

**S-CA** is synthesized from cinnamic acid (CA) via a new, simple and rapid ring-closing reaction method shown in Scheme 1. The yields were calculated based on the last step of the reaction. The typical synthetic procedure involved a novel formation of sultone group with acetic anhydride and concentrated sulfuric acid. Cinnamoyl chloride was synthesized from CA using SOCl_2_ (**1**), and then we obtained 2-cinnamoyl-3-ketobutanoic acid ethyl ester (**2**) by futher reaction with ethyl acetoacetate under strong alkaline conditions. During the synthesis of compound **2**, we observed that the yield of the reaction could be further increased by using NaH instead of sodium ethoxide. Finally, **S-CA** was obtained by ring closure of compound **2** using acetic anhydride and concentrated sulfuric acid.

*Conditions and Regents*: (a) SOCl_2_, reflux, 1 h, 96.7%; (b) NaH, ethyl acetoacetate, THF, ice bath, 2 h, 54.7%; (c) Ac_2_O, H_2_SO_4_, ice bath , 0.5 h, 35.8%.

The most common procedures for the cyclization reaction of sultone derivatives were always complicated by the need for special catalysts and the complexity of their process steps. In contrast to previous syntheses of sultone derivatives, we prepare the targets in a more simple and rapid way. Since no catalysts and cheap reagents were used, this practical approach might be preferred as a relatively “green process”. The new synthetic method expands the scope of methods currently available to introduce the sultone functional group [8]. Meanwhile, this methodology could find applications in the synthesis of naturally occurring sultones. The synthesis method in this article may serve as a basis for a further study of deducing the reaction mechanism. A possible mechanism might be reaction of sulfuric acid with Ac_2_O giving a kind of “mixed anhydride form” followed by nucleophilic attack of the α-C of the enol in compound **2** on this intermediate species. However, a detailed explanation and further studies on the scope and limitations of this atypical reaction needed to be addressed in future work. 

#### 2.1.2. Crystal Structure of **S-CA**

**Figure 1:**
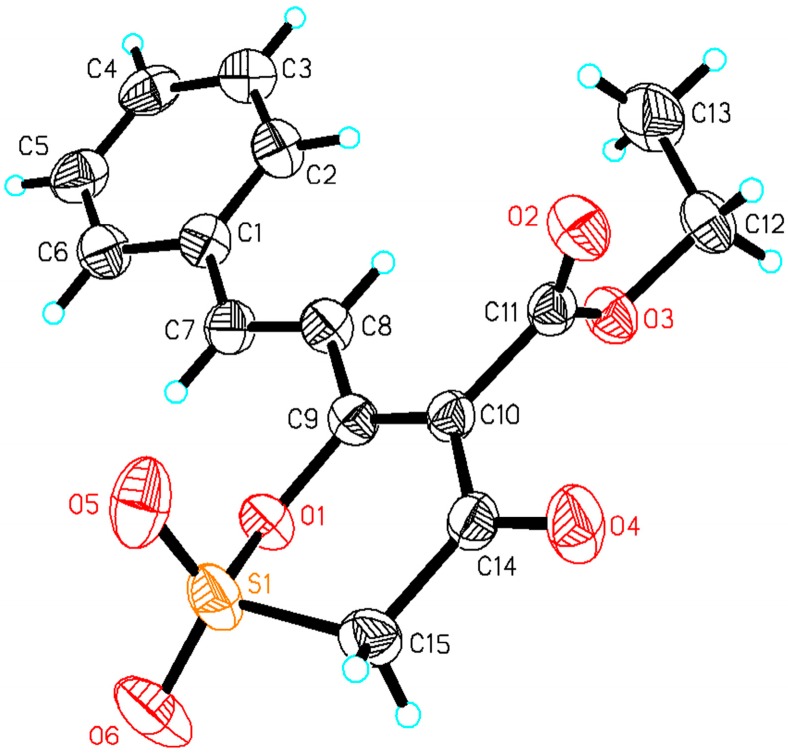
Crystal structure of **S-CA**.

A single crystal of **S-CA** was obtained by recrystallization and its crystal structure was further confirmed by X-ray single crystal diffraction analysis. The structure is shown in Figure 1. Crystallographic data and experimental details for structural analyses are summarized in Table 1.

**Table 1:** Crystallographic data and structure refinement summary for **S-CA**.

Phase	Compound
Molecular formula	C_15_ H_14_ O_6_ S
Formula weight	322.32
T/K	296(2) K
Wavelength/nm	0.71073
Crystal system	Triclinic
Space group	P-1
a/Å	8.5023(2)
b/Å	9.1314(2)
c/Å	10.6477(2)
α (°)	71.512(2)
β (°)	83.356(2)
γ (°)	72.179(2)
V (Å^3^)	746.22(3)
Z	2
F(000)	336
D_calc_ (mg/m^3^)	1.434
Absorption coefficient (mm^−1^)	0.243
θ range/(°)	2.02–27.59
Limiting indices	−10 ≤ h ≤ 11, −11 ≤ k ≤ 11, −13 ≤ l ≤ 13
Reflections collected/unique	7340/3419 [R(int) = 0.0197]
Completeness to theta	99.0%
Data/restraints/parameters	3419/0/199
Refinement method	Full-matrix least-squares on F2
Final R indices[I > 2σ(I)]	R1 = 0.0513, wR2 = 0.1377
R indices (all data)	R1 = 0.0822, wR2 = 0.1586
Goodness-of-fit on F^2^	1.055
Largest diff. peak and hole/(e·Å ×10^−3^)	0.656 and −0.215
CCDC	1052338

### 2.2. Biological Evaluation

#### 2.2.1. Angiogenesis Activity

**Figure 2:**
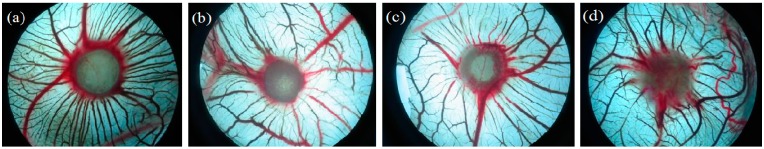
Microvascular proliferation of **S-CA** on CAM (×50). (**a**) Blank control for **S-CA** group, (**b**) Positive control for **S-CA** group, (**c**) 10 μg/egg for **S-CA** group, (**d**) 40 μg/egg for **S-CA** group.

According to the references, sulfonate esters can inhibit angiogenesis [17]. Analogously, the angiogenesis activities of **S-CA** were evaluated by a CAM assay (Figure 2 and Table 2). The model was established according to our previous work [18].

After implantation, the sponge is treated with a stimulator of blood vessel formation in the absence or presence of an angiogenesis inhibitor. Macroscopic observation shows that, the newly formed blood vessels grow radially around the gelatin sponge in the blank control group (Figure 2a). The high survival rate of embryos and normal growth of medium and large vessels (inner diameter > 50 μm) indicates successful modeling and low toxicity of **S-CA** *in vitro* (Table 2). Suppression of small vessels (inner diameter < 50 μm) were recognized as anti-angiogenesis activity. We found **S-CA** could dramatically suppress small angiogenesis in a dose dependent manner on CAM (Figure 2c,d), and the inhibition effect was similarly to that of a positive control (thalidomide, Figure 2b). Based on the above evidence, **S-CA** might serve as an antiangiogenic drug that could enhance the treatment efficacy of cytotoxic chemotherapy.

**Table 2:** Effect of **S-CA** on angiogenesis inhabitation ($\bar{X}$ ± S).

Group	n	Dose (μg/egg)	Large Vessels	Medium Vessels	Small Vessels
**Control**	13	-	8.92 ± 4.80	11.84 ± 3.33	11.6 ± 2.07
**Thalidomide**	13	20	12.4 ± 4.77	12.1 ± 3.92	4.16 ± 3.04 *
**S-CA**	12	10	12.5 ± 6.94	10.33 ± 5.25	5.1 ± 1.22 *
**S-CA**	12	40	12.6 ± 7.68	9.00 ± 4.44	3.5 ± 2.73 *

* *p* < 0.05, large vessels (inner diameter > 100 μm), medium vessels (50 μm < inner diameter < 100 μm), small vessels (inner diameter < 50 μm).

#### 2.2.2. Acute Toxicity

At doses of 10, 20, 30 and 50 mg/kg administered intraperitoneally (i.p.), **S-CA** revealed a regular dose-dependent increase in mortality following acute toxic test. Acute toxicity LD_50_ of **S-CA** is shown in Table 3. According to the results, the LD_50_ of **S-CA** is 25.624 mg/kg by Käber assessment. The mortality rate (0% at 10.0 mg/kg) progressively rose to 100% at the highest dose tested (50.0 mg/kg). The no-observed-adverse-effect level for the i.p. dose was 10 mg/kg. Symptoms such as slow movement decrease in aggressiveness, stopping food intake and weight loss (results not shown) were observed later and at high doses. The acute toxicity data indicated that 10 mg/kg (i.p.) was harmless for the further *in vivo* study in mice.

**Table 3:** Results of **S-CA** on mortality of acute toxicity test in mice.

Group	Mice Number Start/End	Dose (mg/kg)	Death Rate (%)	LD_50_ (mg/kg)	95% CIs
**1**	10/10	10	10	25.624	13.04–50.34
**2**	10/8	20	20		
**3**	10/4	30	60		
**4**	10/0	50	100		

#### 2.2.3. **S-CA**’s Antitumor Activity *in Vivo*

**Figure 3:**
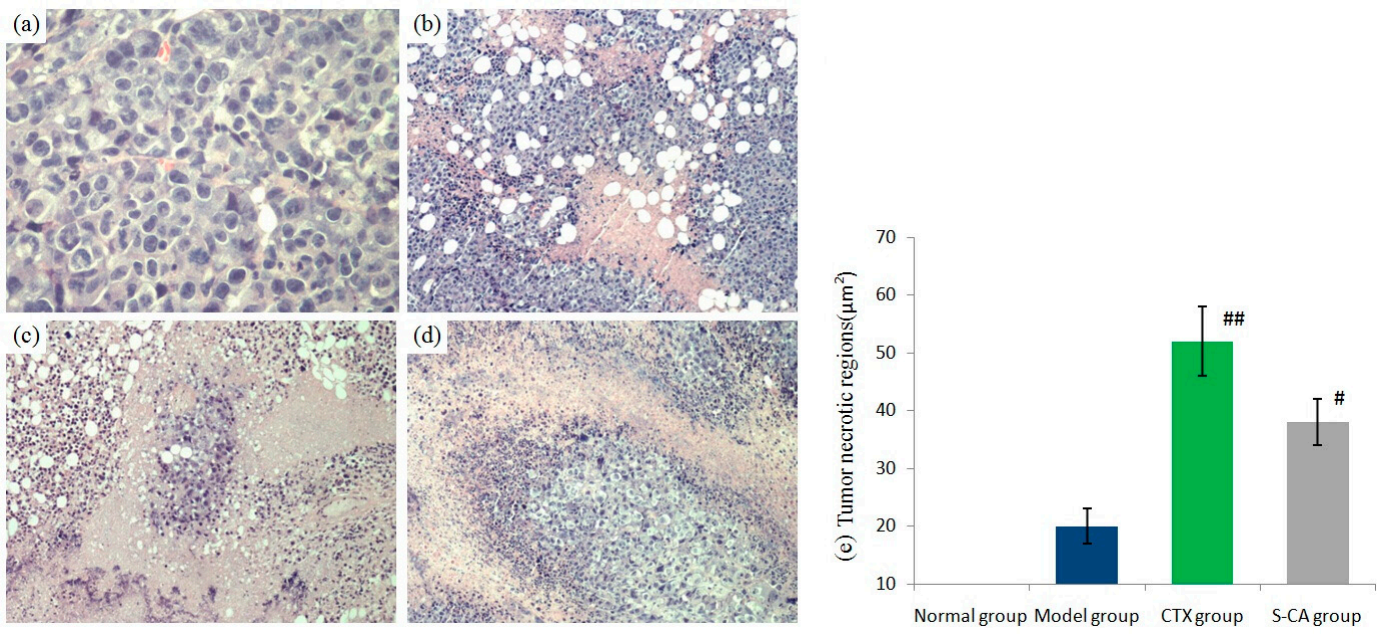
Histopathology of the mice bearing S180 xenografts after the treatment for 15 days. ^#^ compared with model group: ^#^ *p* < 0.05; ^##^ compared with model group: ^##^ *p* < 0.01. (**a**) Model group (400×). (**b**) Model group (100×). (**c**) CTX group (100×). (**d**) S-CA group (100×). (**e**) Tumor necrotic regions with HE staining of model and treatment groups of S180 mice.

Anti-angiogenesis as a way of treating primary tumors and reducing their metastases had been proposed by Folkman in 1971 [19]. Administration of 40 μg/egg **S-CA** led to a significant growth inhibition of the angiogenesis in the CAM model. In this investigation, we screened the **S-CA** antitumor effect in a murine sarcoma S180 model. Treatment with the **S-CA** resulted in marked suppression of tumor weight (Table 4). Compared with the model group, the inhibitory rate of **S-CA** was 44.71% at the doses of 10 mg/kg; meanwhile cyclophosphamide (CTX) caused 63.52% inhibition. The growth of implanted sarcoma S180 tumor in mice could be significantly inhibited by the **S-CA** group (*p* < 0.05). Moreover, HE staining also directly showed that both **S-CA** group and CTX groups’ tumor necrotic regions significantly increased over the model group (*p* < 0.05) (Figure 3). The tumor cells of the model group exhibited a high mitotic index, obvious atypia, whereas tumor cells proliferation of the treated groups were blockaded in some way (Figure 3). Furthermore, the liver indices, which were similar in the normal and **S-CA** treated groups (*p* > 0.05), indicated that **S-CA** did not cause serious toxic effects on the liver system [20]. Spleen is one of the main immune organs; it is responsible for initiating immune reactions in the body. Thus the spleen index directly reflects the status of the immune system [21]. Spleen indices of **S-CA** treated groups were higher than both the normal and CTX groups’. Increases in spleen index suggested that **S-CA** could regulate the immune system of S180 mice [22].

**Table 4:** Antitumor Effects of **S-CA** in S180 Mice (Mean ± S.D.).

Group	Dose (mg/kg)	Mice Number Start/End	Tumor Weight (g)	Inhibitory Rate (%)	Liver Index (100× g/g)	Spleen Index (mg/g)
**Normal**	-	10/10	-	-	4.24 ± 0.54	4.27 ± 0.75
**Model**	-	10/10	0.684 ± 0.416	-	4.51 ± 0.53	5.25 ± 0.94
**CTX**	20	10/10	0.249 ± 0.142 ^#^	63.52%	4.98 ± 0.26	4.50 ± 1.39
**S-CA**	10	10/10	0.342 ± 0.291 ^#^	44.71%	4.81 ± 0.32	6.33 ± 1.51 *

* compared with normal group: * *p* < 0.05; ^#^ compared with model group: ^#^ *p* < 0.05.

## 3. Experimental Section

### 3.1. General Information

Murine sarcoma S180 cells provided by the 302 Military Hospital of China were maintained in RPMI-1640 medium, supplemented with 10% fetal bovine serum (Hyclone, Aurora, ON, Canada), 100 IU/mL Penicillin-Streptomycin (Hyclone), and Non-Essential Amino Acids (Sigma, St.Louis, MO, USA), at 37 °C under humidified air with 5% CO_2_. Male Kunming mice (Beijing Vital River Laboratory Animal Technology Company Limited, Beijing, China) were kept under standard laboratory conditions (tap water, constant room temperature 22 °C). Principles of laboratory animal care were followed and all experiments were carried out in accordance with the “Regulation for the Administration of Affairs Concerning Experimental Animals” [21].

The **S-CA** was synthesized in our laboratory. The purity of **S-CA** was > 98% by HPLC analysis. HPLC-grade methanol and acetonitrile were purchased from Xinkeao Scientific & Technology Co. Ltd (Beijing, China). Other chemicals and reagents were analytical grade and commercially available, and unless otherwise mentioned, used without further purification. Reactions were monitored by TLC using silica gel coated aluminum sheets (Qingdao Haiyang Chemical Co., Qingdao, China) and visualized in UV light (254 nm). ^1^H-NMR and ^13^C-NMR assays were recorded on a Bruker AVANCE 500 NMR spectrometer (Fällanden, Switzerland) and chemical shifts are reported in (ppm). X-ray single crystal diffraction was obtained by using MM007HF Saturn724+ X single crystal diffractometer (Rigaku, Takatsuki-shi, Japan). Melting points are taken with an X-5 micro melting point apparatus and were uncorrected.

### 3.2. Chemistry

#### 3.2.1. Synthesis of **S-CA**

*Preparation of cinnamoyl chloride* (**1**). The mixture of CA (7.4 g, 0.05 mol) and thionyl chloride (SOCl_2_, 10.0 mL) was heated under reflux and stirred for 1 h. The progress of the reaction was monitored by TLC. On completion of the reaction, the extra solvent (SOCl_2_) was removed by reduced pressure distillation, and then the faint yellow solid (8.0 g) was obtained finally. Yield: 96.7%, m.p. 33.8–35.1 °C.

*Preparation of (2Z,4E)-ethyl 2-(1-hydroxyethylidene)-3-oxo-5-phenylpent-4-enoate* (**2**). NaH (0.24 g, 0.10 mol) was added to ethyl acetoacetate (5.7 mL, 0.04 mol) was added and the mixture was stirred under ice bath. After air bubbles production ceased, cinnamoyl chloride (6.6 g, 0.04 mol) dissolved in THF was added drop wise and the reaction was kept under ice bath for 2 h. The reaction mixture was acid treated with 5% HCl and then extracted by ethyl acetate. After drying the organic layer over anhydrous Na_2_SO_4_ and evaporating the solvent under vacuum, the title compound was finally obtained as a semi-solid (5.7 g, 0.022 mol). Yield: 54.7%. ^1^H-NMR (CDCl_3_) (ppm): 1.41 (t, 3H, *J* = 14.0 Hz, -CH_3_), 2.43 (s, 3H, CH_3_), 4.36 (q, 2H, *J* = 14 Hz, -CH_2_-), 7.43 (d, 1H, *J* = 15.5 Hz, =CH-), 7.44–7.46 (m, 3H, Ar-H), 7.56–7.57 (m, 2H, Ar-H), 7.82 (d, 1H, *J* = 15.5 Hz, -CH=). ^13^C-NMR (CDCl_3_) (ppm): 14.3 (-CH_3_), 27.4 (-CH_3_), 60.4 (-CH_2_), 108.5, 120.9, 128.5, 128.9, 130.5, 135.0, 143.1, 167.3 (-COO-), 182.0 (-C=O), 199.7 (HO-C=).

*Preparation of (E)-ethyl 4-oxo-6-styryl-3,4-dihydro-1,2-oxathiine-5-carboxylate 2,2-dioxide* (**3**). Compound **2** (5.0 g, 0.02 mol) was dissolved in acetic anhydride (25 mL) under ice bath cooling, and concentrated sulfuric acid (0.6 mL) was added dropwise over 0.5 h. After completion of this reaction, the reaction mixture was poured into ice-water and the orange-red precipitated solid was obtained. The crude product was recrystallized from ethanol to afford the yellow crystal (1.4 g). The purity of synthesized **S-CA** was more than 98%. The structure of **S-CA** was determined by ^1^H-NMR, ^13^C-NMR, HMQC, and X-ray single crystal diffraction analysis. Yield: 35.8%, m.p. 128.7–129.1 °C. ^1^H-NMR (CDCl_3_) (ppm): 1.43 (td, 3H, *J* = 1.5, 14.0 Hz, -CH_3_), 4.42 (s, 2H, -CH_2_-), 4.46 (qd, 2H, *J* = 1.5, 14 Hz, -CH_2_-), 7.05 (dd, 1H, *J* = 3.5, 15.5 Hz, -CH=), 7.46–7.48 (m, 2H, Ar-H), 7.59–7.60 (m, 3H, Ar-H), 7.73 (dd, 1H, *J* = 3.5, 15.5 Hz, =CH-). ^13^C-NMR (CDCl_3_) (ppm): 14.2 (-CH_3_), 59.2 (-CH_2_), 62.7 (-CH_2_), 115.4, 116.0 (-CH=), 128.9 (Ar-C), 129.2 (Ar-C), 131.7 (Ar-C), 134.0, 144.4 (=CH-), 162.6, 163.0, 179.8.

#### 3.2.2. Crystal Structure

The crystal and molecular structure has been determined by X-ray single crystal diffraction. The coordinate and anisotropy parameters of non-hydrogen atoms were refined with a full-matrix least-squares procedure to R_1_ = 0.0513, wR_2_ = 0.1377, (∆p) max = 0.656 × 10^−3^ e nm^−3^, (∆p) min= −0.215 × 10^−3^ e Å^−3^. The structure was solved by using the program SHELXS-97 and Fourier difference techniques and refined by full-matrix least-squares method on F^2^ using SHELXL-97. Details of the data collection and refinements of the title compound are given in Table 1 and Figure 1. All non-hydrogen atoms were refined anisotropically, whereas the hydrogen atoms were generated geometrically. Parameters in CIF format are available as Electronic Supplementary Publication from Cambridge Crystallographic Data Centre. CCDC 1052338 contains the supplementary crystallographic data for this paper. These data can be obtained free of charge via http://www.ccdc.cam.ac.uk/conts/retrieving.html.

### 3.3. Bio-Evaluation Methods

#### 3.3.1. Angiogenesis Assay

Fertilized White Leghorn chicken eggs (50–65 g), provided by the Chinese Academy of Agricultural Sciences, were placed in an incubator as soon as embryogenesis started and were kept under constant humidity of 65% at 37 °C. Here we present a method for the antiangiogenesis in the chick embryo chorioallantoic membrane (CAM) based on the implantation of a gelatin sponge on the top of the growing CAM on day 7 of development. On day 7, under sterile conditions, a square window was opened on the shell and physiological saline (0.1 mL) was injected in to detach the shell membrane. Gelatin sponges were implanted, respectively. The control group was treated with acetone (10 μL). Moreover, 1 mm sterilized gelatin sponges carrying the **S-CA** dissolved in acetone at 10 and 40 μg/egg were implanted on the smaller vessels part of CAM. The window was sealed with sterile adhesive and the eggs were returned to the incubator for a further 48 h. Then, the tapes were removed and the entire CAM was detached after tissue fixation with methanol/acetone (1:1, v/v). We use computer-assisted tracking of images to obtain absolute values for the number of microvessels. Quantitative evaluation of the angiogenic response, expressed as microvessel density, can be obtained by applying a morphometric method of “point counting” on histological CAM sections. Data was analyzed using *t*-test of statistics analysis system, the values were expressed as mean ± sd of 6 observations and *p* < 0.05 was considered significant. 

#### 3.3.2. Acute Toxicity Test

**S-CA** was further investigated for its approximate LD_50_ and 95% confidence interval in mice. Male Kunming mice (Beijing Vital River Laboratory Animal Technology Company Limited, Beijing, China) weighing 19.2–21.2 g, were randomly divided into five groups per ten individuals matched in weight and size. In the models, group I received solvent (bean oil) and this group was used as controls. Then the other four groups were given lumbar injection of **S-CA** in doses of 10, 20, 30, 50 mg/kg respectively. The general behavior of the mice was observed continuously for 1 h after the treatment and then intermittently for 4 h and thereafter over a period of 24 h. At the end of this period mortality was recorded for each group. The acute toxicity was evaluated by the median lethal dose (LD_50_) and 95% confidence interval which were calculated by Käber assessment. The summary of **S-CA** acute toxicity expressed as LD_50_ is shown in Table 2. The mice were further observed for up to 7 days following treatment for any signs of toxicity and deaths and the latency of death.

#### 3.3.3. Antitumor Activity *in Vivo*

S180 cells were harvested and washed three times with RPMI-1640 medium. The cells were pelleted by brief centrifugation at 800 r/min for 10 min. The supernatant was aspirated, and the cells were resuspended in normal saline at a density of 2 × 10^7^ cells/mL. Male Kunming mice were subcutaneously implanted with 2 × 10^6^ cells/mouse on the left flank (day 0). Twenty-four hours after inoculation, 30 tumor bearing mice were randomly divided into model group, CTX group, **S-CA** group (n = 10), respectively. Another 10 mice without any treatment were set as normal group. **S-CA** was continuously administrated via intraperitoneal injection, every other day for 15 days. **S-CA** groups received 10 mg/kg of body weight, the CTX group received CTX (20 mg/kg), model and normal groups were treated with bean oil, respectively. Twenty-four hours after the last administration, the mice were sacrificed and the solid tumors, livers and spleens were excised and weighed. The inhibition rate (IR) of tumor growth was calculated by the following formula: IR (%) = [(A − B)/A]/100, where A is the average tumor weight of the model group, and B is that of the treatment group mice. The index of livers was calculated as W_l_/W_m_, where W_l_ was the average livers weight (g) of each group and W_m_ was the average mouse body weight (g) of each group. The index of spleen was calculated as W_s_/W_m_, where W_s_ was the average spleen weight (mg) of each group and W_m_ was the average mouse body weights (g) of each group.

At the end of the experiments, a thorough necropsy was carried out immediately after the mice were sacrificed. Samples of the tumors and the following organs, liver and spleen were fixed in 10% neutral buffered formalin, embedded in paraffin, sectioned at 4 mm, and processed according to the hematoxylin and eosin (HE) staining protocol. The stained tissues were observed under a light microscope. Quantitative analysis was made in a blinded manner under a light microscope. Computer-assisted image tracking was used to calculate the positive tumor areas. The results were regarded as the mean ± SD of eight different sections.

## 4. Conclusions

In summary, a simple method for the synthesis of the sultone derivative **S-CA** and its characterization by ^1^H-NMR, ^13^C-NMR, HMQC and X-ray single crystal diffraction analysis are reported. In addition, **S-CA** exhibited selective antiangiogenesis on CAM and potent antitumor effect against S180 model. While there have been no major breakthrough in recent years in synthesis routes to this functional group, our study can be regarded as a simple method for the synthesis of the sultone derivative **S-CA**. Moreover, sultone derivatives are used widely in many other fields, including industrial applications, for their biological properties and the current mechanistic research into the role of β-sultones in olefin sulfonation. Many materials containing nucleophilic functionalities have been modified by propane-sultone, producing fungicides, fire resistant polymers, lubricating oil additives and emulsifying agents. 1,3-Propanesultone is used as starting material in the synthesis of many electroplating intermediates and also used for the synthesis of a variety of sensitizing dyes and surfactants. The new synthesis route extends the ring closing reaction of sulfonic acid lactone derivatives method and has potential applications in general organic synthesis, natural product synthesis and some other research fields as well.

Based on the current study, **S-CA** represents a new hit for the biological activities of sulfonate units and should be further studied for antiangiogenesis in anticancer drug discovery. The difference of toxicity in the biological evaluation on CAM and an acute toxicity test confirmed the potentially pharmacologically interesting properties of **S-CA**. Our completed work lays the foundation for further research on the anti-tumor mechanism of sultone derivatives. A further study to acquire information concerning its target organs and pharmacological activity is still in progress.

## Supplementary Materials

Supplementary materials can be accessed at: http://www.mdpi.com/1420-3049/20/03/4307/s1.
