# Supplementary material for: New Synthesis Method for Sultone Derivatives: Synthesis, Crystal Structure and Biological Evaluation of S-CA
Source: Molecules. 2015 Mar 6;20(3):4307–18. doi: 10.3390/molecules20034307 (PMC6272555; doi:10.3390/molecules20034307)
Supplement: Supplementary file 1 [file molecules-20-04307-s001.pdf]

Supplementary Materials

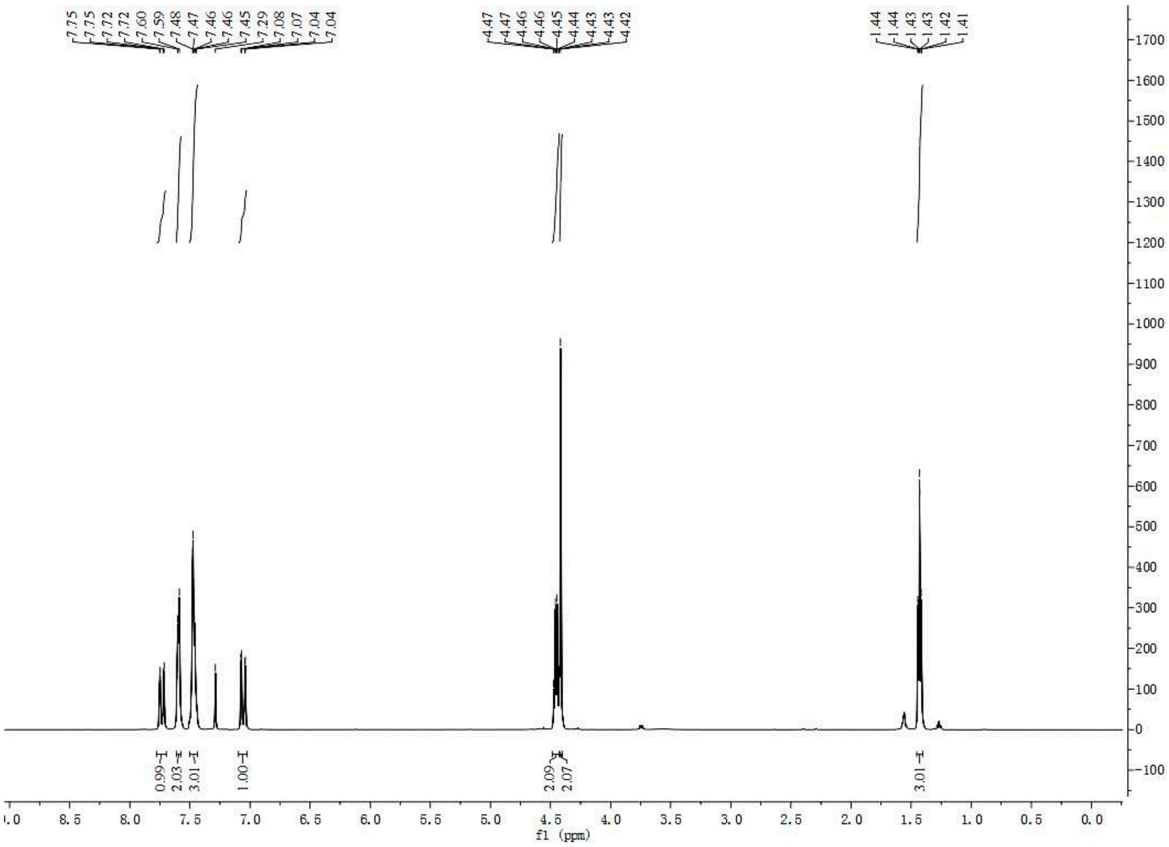

Figure S1. ^{1}H-NMR spectrum of **S-CA**.

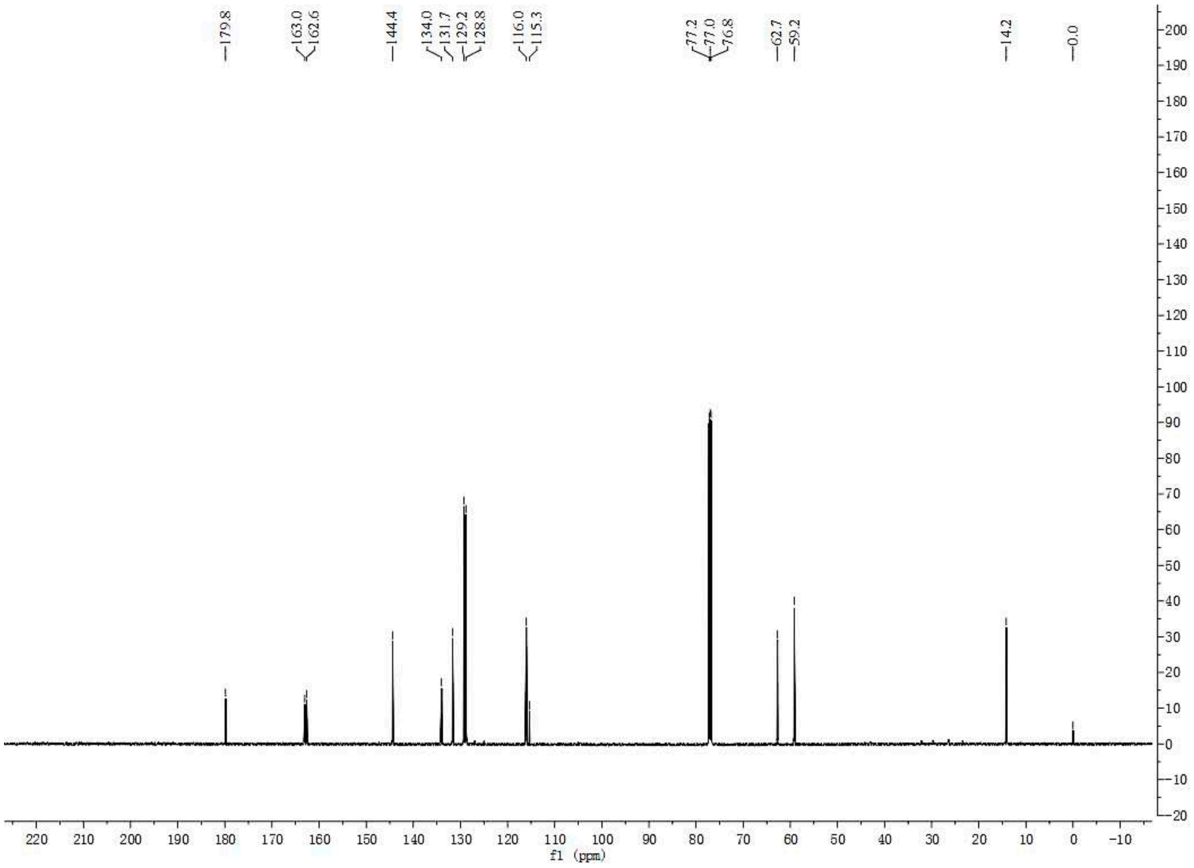

Figure S2. ^{13}C-NMR spectrum of **S-CA**.

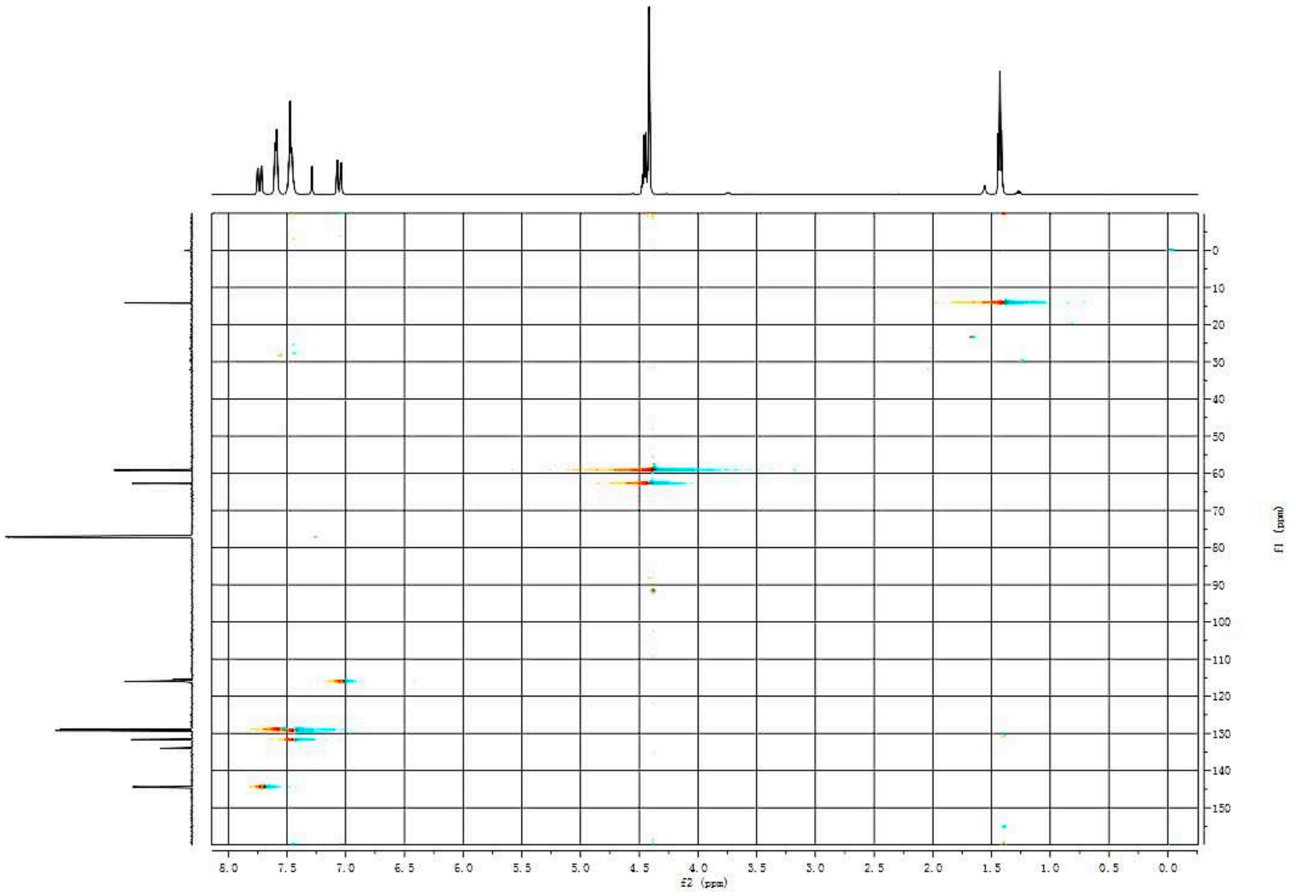

Figure S3. HMQC spectrum of **S-CA**.
